# Purification and biochemical characterization of l-glutaminase from *Aspergillus oryzae* with potential biotechnological applications in synthesis of l-theanine and as antitumor agent

**DOI:** 10.1038/s41598-025-21904-8

**Published:** 2025-10-21

**Authors:** Hamed M. El-Shora, Sally M. Metwally, Nahla T. Elazab, Widad M. Al-Bishri, Gharieb S. El-Sayyad, Reyad M. El-Sharkawy

**Affiliations:** 1 Department of Botany, Faculty of Science, Mansoura University, Mansoura, 35511 Egypt; 2 Department of Botany and Microbiology, Faculty of Science, Tanta University, Tanta, Egypt; 3 Department of Biology, College of Science, Qassim University, Buraydah, Qassim 51452 Saudi Arabia; 4 Department of Biological Sciences, College of Science, University of Jeddah, 80327 Jeddah, Saudi Arabia; 5 Medical Laboratory Technology Department, Faculty of Applied Health Sciences Technology, Badr University in Cairo (BUC), Cairo, Egypt; 6 Drug Microbiology Lab, Drug Radiation Research Department, National Center for Radiation Research and Technology (NCRRT), Egyptian Atomic Energy Authority (EAEA), Cairo, Egypt; 7 Botany and Microbiology Department, Faculty of Science, Benha University, Benha, 13511 Egypt

**Keywords:** *Aspergillus oryzae*, l-glutaminase, Purification, Trehalose, l-theanine, Deamidation, Glutelin, Enzymes, Cancer

## Abstract

Fungal glutaminase is of great importance in different industries fields. Thus, searching for a significantly catalytic l-glutaminase (Glut) with appraising its biochemical properties and biotechnological applications are the main purposes of this work. The Glut from *Aspergillus oryzae* was purified with a specific activity 258.33 (U/mg of protein), 215-fold and 36.47% yield. The molecular weight of the purified enzyme was 65 kDa. The purified enzyme exhibited remarkably improved resistance toward higher temperature in the presence of an exogenous trehalose. The enzyme had a greater affinity towards l-glutamine, l-cysteine, l-proline and l-lysine than l-valine, and l-glycine. The essentiality of arginine, tryptophan, histidine, and cysteine residues in the catalysis process of l-glutaminase was determined. The application of biocatalyst in the reaction mixture has not only remarkably increased l-theanine concentration but also has enhanced glutamic acid production in the presence of 10% compared with 15% NaCl. The deamidation of water insoluble *Zea* or rice glutelin was approximately 65% and 83% after 44 h by the enzymatic treatment. The purified Glut displayed remarkable antitumor activities against lung (A549), liver (HepG2) and human breast (MCF-7) carcinoma. Thus, l-Glut from *A. oryzae* has the potential to be used in food application and in treatment of various cancer cells.

**Supplementary Information:**

The online version contains supplementary material available at 10.1038/s41598-025-21904-8.

## Introduction

The extensive applications of microbial enzymes in agriculture, cosmetics, chemical, and food industries, along with their eco-friendly and cost-effective nature, are key drivers behind the rapid expansion of the enzyme biotechnology sector ^1–5^. l-glutaminase (Glut, EC 3.5.1.2) is an amidohydrolase which mechanistically catalyzing the deamidation of l-glutamine to l-glutamate. Although, the l-glutaminase is ubiquitous biocatalyst among different categories of plants and microorganisms (bacteria, fungi and yeasts), fungi are considered as the most promising l-glutaminase producers, particularly *Penicillium* sp., *Fusarium* sp., *Acremonium* sp., *Trichoderma* sp., *Aspergillus oryzae* and *Aspergillus flavus *^4,6–8^. l-glutaminase obtained from different sources has been purified to homogeneity, and its biochemical properties have been extensively studied ^4,6,7^. Fungal l-glutaminase has garnered significant attention due to its potential roles in cancer cell chemotherapy, the production of l-glutamic acid and l-theanine, and various other biotechnological applications ^9,10^. It was found that theanine induced cell death in the tumor cells, but not in the normal cells. A glutamine-based biosensor was employed in detecting the level of ammonia in a biological samples ^1,11^. It is useful in the production of unique non-protein amino acid, l-theanine (γ-glutamylethylamide). Such compound is characterized by its potential anti-cancer, anti-hypertensive, neuro-protective; however it cannot be synthesized in the human body, hence the production of l-theanine in the presence of l-glutamine and ethylamine using fungal enzymes should be considered in the future studies ^12^.

To meet the growing demand for l-theanine, various production methods have been developed, including chemical synthesis, extraction from tea leaves, enzymatic synthesis, and microbial fermentation ^13–16^. Fan et al. ^17^ reported that l-theanine could influence invasion, migration, and enhance adhesion of carcinoma cells both in vivo and in vitro. The synthesis of l-theanine has been achieved using *Pseudomonas nitroreducens* l-glutaminase. Additionally, l-glutaminase from *Trichoderma koningii* catalyzes the reaction between ethylamine and l-glutamine to produce l-theanine ^12^. l-glutaminase performs an essential role in improving delicious taste of fermented food products through the biocatalytic activity of this enzyme, enhancing the glutamic acid contents of fermented food ^10,18,19^. l-glutamic acid production can be obtained from biomass protein by either enzymatic or chemical hydrolysis. The difficulty of protein solubilization in aqueous solution, which may be due to the hydrophobic nature and the disulphide bonds among protein subunits, make its necessary for chemical and enzymatic hydrolysis in order to enhance amount of liberated glutamic acid. The enzymatic hydrolysis process is preferred over chemical methods, which is attributed to its eco-friendly, and low cost ^20–22^.

The cereals-based food industries are moving beyond the normal processing way. For this instance, numerous attempts have been performed for investigating the potentiality of adding significant nutritional value. Cereals containing proteins are considered as an important source for valuable essential amino acids, accounting for approximately 8–13% protein concentration in milled cereals. Such proteins and its amino acids can perform several functions including hypocholesterolemic and hypoallergenic. In addition, the manufacture of food materials requires a variety of specific properties like emulsifying abilities, gelling and foaming; however, the presence of high glutamine-asparagine content resulting in the aggregation of cereals proteins ^23–25^. Hence, in order to improve the nutritional value of cereals, the deamidation of food proteins is applied for its ability to enhance the quality, solubility, stability and other functional characters of food protein products. The enzymatic protein deamidation is desirable technique for protein modification if compared to chemical methods. The deamidation by glutaminase is one of the most significant tools used for water-insoluble protein deamidation ^24,26,27^. Unfortunately, few studies have been done on the enzymatic deamidation of cereals proteins.

The nucleotides and amino acid biosynthesis in a living cell is commonly controlled by the level of l-glutamine in the cell. It is deemed to be an obligate nitrogen donor for such synthetic process. In addition, it plays a role as major substrate for mitochondria, production of NADPH and preserving the mitochondrial membrane ^28–30^. The tumor cells are unable to synthesize _L_-glutamine; however, glutaminolysis (metabolism of glutamine) is a key distinctive character for malignant cells. Hence, I-glutaminase became important to cause a depletion of l-glutamine and consequently inhibits glutamine-dependent tumor cells ^6,10,28^.

The objectives of this work were focused on the purification of l-glutaminase from *Aspergillus oryzae* with promising catalytic proficiency toward l-glutamine, evaluating its biochemical characters and investigating its biotechnological applications by accentuating on l-theanine and _L_-glutamic acid production, deamidation of glutelin from *Zea* and rice as well as explore its cytotoxic efficiency against various carcinoma cell lines.

## Materials and methods

### Materials and chemicals

2,3-butanendione (BD), N-bromosuccinimide (NBS), diethyl pyrocarbonate (DEPC), N-ethylmaleimide (NEM), and dichlorofluorescin diacetate were donated from Sigma Chemical Co. (Cairo, Egypt). Other chemicals of analytical grade include l-glutamine, l-valine, l-methionine, l-proline, l-glycine, _L_-lysine, _L_-cysteine, ethylamine, were purchased from Sigma Aldrich. All other reagents and chemicals utilized in the current research were of analytical grade. A549 cell lines, MCF-7 (human breast cancer cells), and HepG2 (human hepatocellular carcinoma cells) were purchased from ATCC via holding company for biological products and vaccines (VACSERA), Cairo, Egypt.

### Fungal isolates, culture conditions and qualitative screening for l-glutaminase producing isolates

The most potent fungal strain was selected from twenty isolates that collected from soil samples of Benha Governorate, Egypt. Briefly, soil specimens were collected in aseptic polyethylene bags. The isolation was performed using selective minimal agar media (SMA) containing % (glucose 0.2; MgSO_4_.7H_2_O 0.05; KH_2_PO_4_ 0.15; FeSO_4_.7H_2_O 0.01; KCl 0.05; agar 1.5) supplemented with 1% l-glutamine. 1 mL of soil sample dilution (10^–4^) was inoculated into modified glutamine salt agar (GSA; SMA + 1% l-glutamine) plates and were incubated for 4 days at 28 °C.

The capability of the fungal isolates for l-glutaminase production was tested using the rapid plate screening assay method ^25,31^. GSA was prepared with phenol red (0.009%, w/v) as indicator dye and the pH was adjusted to 6.0. All the plates were inoculated with a spore suspension (1 × 10^6^ spore/mL) of the investigated fungal isolates and incubated for 4 days at 28 °C. In parallel, the culture media with NaNO_3_ as a sole nitrogen source was used as control. After incubation, the color change from yellow to pink is considered as positive observation. Zone index (ZI) was estimated by dividing the inner and outer diameter^32^.

### Quantitative determination of l-glutaminase activity

l-glutaminase activity was quantitatively assayed according to Awad et al. ^6^ in the selected overproducing I-glutaminase isolate, based on the maximum zone indices. A spore suspension (1 mL) of 1 × 10^6^ spore/mL was introduced into 250 mL Erlenmeyer flasks containing 50 ml of selective mineral broth supplemented with 1% l-glutamine and phenol red (0.009%, w/v). The initial pH was adjusted to 6.0. After incubation at 28 °C for 4 days at 150 rpm, the fungal mycelium was removed by filtration. The filtrate was centrifuged at 4 °C for 15 min at 10,000 x*g*. The resulting supernatant was used for enzyme activity analysis and preserved at – 20 °C for further work ^33,34^.

### Assay of I-glutaminase activity and protein determination

The l-glutaminase activity was determined based on the amount of released ammonia as mentioned by Nesslerization method ^35^. The reaction mixture contains 50 µl of the enzyme preparation and 0.5 mL of glutamine (0.2 M) in 450 µl Tris–HCl buffer (pH 7.0, 50 mM). The reaction mixture was incubated at 37 °C for 15 min. The reaction was quenched by of trichloroacetic acid (0.5 mL, 10%), centrifuged for 10 min at 10,000 x*g*. The supernatant, distilled water and Nessler’s reagent were respectively mixed for 10 min in a proportion of 1 mL, 1 mL and 250 µl. Blank of reaction mixture without enzyme was achieved under the same conditions as baseline. Using a standard curve constructing by using ammonium sulfate, the enzyme activity was spectrophotometrically determined at 450 nm. One unit of l-glutaminase was termed as the amount of enzyme which releases 1 µmol of ammonia per mg enzyme per minute. The enzyme protein concentration was determined according to the recent publication ^36^.

### Molecular identification of the promising I-glutaminase producing isolate

The purified strain with the overproducing l-glutaminase was identified based on molecular sequence analysis ^37,38^. The molecular confirmation was performed based on the entire sequence of internal transcribed spacers (ITS) region ^5,39,40^. The genomic DNA was extracted as mentioned by the following reference ^41^. Using the gDNA as template, the ITS region was amplified using primer sets of ITS1 (5’-TCC GTA GGT GAA CCT GCG G-3’) and ITS4 (5’-TCC TCC GCT TAT TGA TAT GC-3’) in a polymerase chain reaction (PCR). The PCR reaction includes 2 × PCR master mixture (AlphaDNA Co, Canada), 1 µl of reverse and forward primers, and 1 µl of gDNA. The PCR was carried out by Solgent EF-Taq, PCR Machine name: 9700(ABI), MJ research thermal cycler (USA). The PCR was programmed to 95 °C for 3 min, followed by 35 cycles of 95 °C for 30 s, 50 °C for 30 s and 72 °C for 90 s and a final extension 72 °C for 5 min. The purified PCR product was sequenced by the same primer sets. The obtained sequence was matched using BLAST tool with the ITS sequences on the NCBI database. For phylogenetic analysis, multiple sequence alignment by ClustalW muscle algorithm was carried out using MEGA portal (version 10.0). The phylogenetic tree was created by the neighbor-joining method using 1000 bootstrap repeats/branch analysis.

### l-glutaminase purification of l-glutaminase

The mycelia-free supernatant was purified by using ammonium sulphate powder (30–90% saturation). The crude l-glutaminase was first brought to 30% saturation using solid ammonium sulphate with gentle agitation at 4 °C for 24 h. The precipitated proteins were collected by centrifugation for 20 min at 8,000 × *g*. The resulted pellet was dissolved in 10 ml of the phosphate buffer. The supernatant was applied again with ammonium sulphate to achieve 40, 50, 60, 70, 80 and 90% saturation and the correlation coefficient was 0.997. The precipitate was dialyzed at 4 °C in acetate buffer (0.1 M, pH 5.0) for 24 h. The obtained concentrated enzyme was subject to DEAE-cellulose column (2 × 15 cm, Sigma) which had been equilibrated with 0.1 M acetate buffer (pH 5.0). The fractions were combined and eluted using the same buffer with a linear gradient of sodium chloride (0.0–1.0 M) at a flow rate of 12 ml/h, and 2 ml fractions were collected. Sephadex G-200 column (2 × 20 cm, Sigma) was equilibrated with acetate buffer (0.1 M, pH 5.0), loaded with the pooled fractions, and subsequently the elution was performed using the same buffer with a linear gradient of sodium chloride (0.0–1.0 M) at a flow rate of 12 ml/h, and 2 ml fractions were collected. The l-glutaminase activity and the protein concentration were determined as mentioned before.

### Sodium dodecyl sulphate polyacrylamide gel electrophoresis (SDS-PAGE)

Glutaminase’s molecular mass was determined using one-dimensional SDS-PAGE, following Laemmli’s method ^42^. The analysis was performed with a 5% stacking gel (pH 6.8) and a 12% resolving gel (pH 8.8). Under reducing conditions SDS-PAGE samples were prepared by mixing 20 µl of the purified enzyme with 5 µl of buffer containing 0.5 M Tris–HCl (pH 6.8), 4% SDS, 10% glycerol, and 10% 2-mercaptoethanol, followed by incubation at 100 °C for 5 min. The prepared samples were then loaded onto the gel and electrophoresed under reducing and denaturing conditions at a constant voltage of 70 V. The gel was stained overnight with Coomassie Blue and subsequently de-stained using a destaining solution, after which protein bands were visualized.

A range of standard protein markers was used to estimate the molecular weight. The markers were chosen with molecular weight between 30 and 97 kDa. The molecular weight of purified l-glutaminase was determined by using markers of low molecular weight, namely carbonic anhydrase (30 kDa), ovalbumin (43 kDa), bovine serum albumin (66 kDa) and phosphorylase (97 kDa).

### Biochemical characterization of ***A. oryzae***l-glutaminase

#### Reaction pH, temperature, pH stability, thermostability and substrate affinity

The biochemical properties of the purified *A. oryzae* l- glutaminase were determined at various pH values in the range of 3.0–9.0 using (100 mM of Na acetate/phosphate buffer (pH 4–6)/Tris–HCl buffer (pH 7–9); various temperatures (20–60°C); various amino acids (0.2 mM) substrates (l-glutamine, l-valine, l-methionine, l-glycine, l-lysine, l-cysteine, l-proline,) and the enzyme activities were assayed under standard assay conditions. The stability of l- glutaminase was investigated by pre-incubating the enzyme without substrate, at different pH values (3.0–9.0). The thermostability of the enzyme was carried out by preincubating the enzyme preparation at different temperatures (20–60°C) for 15 min in the presence or absence of 10 mM trehalose. The residual activities were evaluated in standard conditions.

#### Stability of l-glutaminase in presence of different metal ions

The stability of l-glutaminase in presence of metal ions was assessed by pre-incubating the enzyme in the presence of different metal ions as chloride salts (KCl, MgCl_2_, CaCl_2_, CoCl_2_, CuCl_2_, AlCl_3_) at both 5 mM and 10 mM in 0.1 M phosphate buffer (pH 7.0) for 1 h at 35 °C. The biocatalytic activity in the absence of metal ions was determined, referenced as 100% and the residual activities were determined in absence of metal ions.

#### Susceptibility towards active group reagents

The principle of this experiment is based on the idea that if the enzyme activity is inhibited in presence of any of the reagents butanedione-BD, N-bromosuccinimide-NBS, diethyl pyrocarbonate-DEPC, and N-ethylmaleimide-NEM in the assay medium, it will suggest that the corresponding amino acid residue plays a critical role in the enzyme’s catalytic function. On the other hand, if the enzyme remains unaffected, it indicates that the specific amino acid residue is not essential for the enzyme’s activity.

Therefore, _L_-glutaminase activity was assessed in the presence of these various group reagents. The assay was performed by preincubating the purified biocatalyst with each reagent alone in 0.1 M phosphate buffer (pH 7.0) at various concentrations (2–10 mM). The mixture was then incubated for 20 min at 37 °C. In parallel, the reaction mixture without adding chemicals was performed under the same conditions and the enzyme activity was assayed ^5,33^. The remaining activity of I-glutaminase was calculated, relative to the original activity which represents 100%. The remaining (relative) activity = (Final activity/Original activity) × 100.

#### Kinetic parameters

The V_max_ and K_m_ of I-glutaminase were estimated from Lineweaver-Bürk plot using _L_-glutamine at concentrations from 0.25 to 12 mM under optimal assay conditions (pH 7.0 at 35 °C for 1 h) ^43^.

### Biotechnological applications of I-glutaminase

#### Production of I-theanine

The production of I-theanine was performed in the presence ethylamine, I-glutamine, and the purified I-glutaminase from *A. oryzae* according to the following work ^1^ with slight modifications. The reaction was carried out by incubating 0.2 M I-glutamate, 1.3 M ethylamine, 0.01 M ATP, 0.05 M MnCl_2_, with different concentrations of _L_-glutaminase (1–10 units) in phosphate buffer (pH 7.0, 0.5 M) for 48 h at 35 °C with shaking at 150 rpm. The reaction was stopped through dipping of the test tube for 3 min in boiling water. After centrifugation for 10 min at 10,000 × *g* at room temperature, the concentration of I-theanine produced was assayed according to the following citations ^1,4,44^. In brief, aliquot of I-theanine solution was mixed with 1 mL of ethanolic ninhydrin in 1 mL acetate buffer (pH 5.4) for 15 min at 100 °C. The absorbance was determined at 570 nm and the concentration was measured based on the standard curve obtained by various I-theanine concentrations (5–100 mM).

#### Deamidation of glutelin from *Zea* and rice using I-glutaminase

*Zea* or rice flour was purchased from a local market in Benha, Egypt. For defatting the flour, samples of each flour were separately mixed with continuous stirring at Lab temperature for 1 h with hexane (1:5 w/v). The flour was then recovered by a Buchner funnel, air dried under a hood, followed by pass through a sieve of 75 µm. For preparation of both flour glutelin, the defatted preparations were undergo sequential extraction of the albumin and globulin using distilled water (400 mL) and NaCl (400 mL, 5%), respectively. After extraction, the glutelin and prolamin fractions were extracted from the developed residues using NaOH (100 mM, 1 h) and 70% ethanol at 25 °C using the procedure of Agboola S. et al., ^26^ with minor modifications, consecutively. The extraction procedures were repeated twice in order to eliminate the protein. After isoelectric precipitations of the albumin, globulin, glutelin and prolamin fractions and washing with distilled water, the preparations were then dialyzed for 24 h against distilled water at 4 °C, followed by freeze dried and stored at 4 °C. For deamidation process, the reaction mixture includes 10 mg/mL glutelin and I-glutaminase (0.5 unit) in phosphate buffer (pH 7.0, 0.1 M) was incubated for different time intervals (0–60 h) at 30 °C. In parallel, controls containing glutelin fractions of *Zea* or rice, without enzymatic preparation were runs under the same standard conditions. The ammonia released during I-glutamine deamidation was determined using Nesslerization method^25,35^. Deamidation degree (%) is calculated by relative ratio of the amount of ammonia released by I-glutaminase to the total ammonia liberated during the treatment process of proteins for 72 h by 2M HCl at 110 °C ^45^.

#### Production of I-glutamic acid

The I-glutamic acid production using I-glutaminase was investigated according to Wakayama M. et al., ^46^ with slight modifications. A model system containing 10g soybean protein, crude protease, adequate amount of *A. oryzae* I-glutaminase and NaCl with a final concentration of 10% or 15%. The reaction was performed at 30 °C for 70 h and the concentration of glutamic acid produced was assayed.

#### In vitro antitumor activity of I-glutaminase against various cell lines

The cytotoxic efficiency of the purified I-glutaminase was evaluated against human lung carcinoma (A549), Liver (HepG2), human breast carcinoma (MCF-7) cell lines and on human normal cell line (Wi 38) as control. The assay was performed according to Ebadi et al. ^47^ using MTT (3-[4,5-dimethhylthiazol-2-yl]-2,5-diphenyltetrazolium bromide). The efficiency of I-glutaminase as antitumor activity was represented by median growth inhibitory concentration (IC_50_). The test was carried out in 96-microtitre plate (Sigma Aldrich, USA), each well was seeded with 1 × 10^4^ in a total volume 100 µl growth medium. The cells were grown and maintained in Dulbecco’s Modified Eagle’s (DEMEM) medium supplemented with 10% (v/v) fetal bovine serum (FBS) and 1% penicillin/streptomycin. The cells were treated with various concentrations of I-glutaminase (20–100 µg/mL), compared to doxorubicin as a chemo-therapeutic agent and incubated at 37 °C for 48 h in a humidified incubator with 5% CO_2_. The medium was then aspirated and 5 mg/mL MTT was added to each well, subsequently incubated for 3 h. The formazan crystals were dissolved in 100 mL acidified isopropanol and read at 570 nm using ELISA reader (Bio-Rad micro plate reader, Japan). Cell viability percentage was determined by the following equation:

Cell viability (%) = $$\frac{{\text{OD}}_{\text{T}} }{{\text{OD}}_{\text{C}}} \times 100$$

Where ${\text{OD}}_{\text{T}}$ is the optical density of treated cells and ${\text{OD}}_{\text{C}}$ is the optical density of control cells. Each experiment was carried out in triplicate.

#### Measurement of intracellular ROS generation

Following incubation periods of 24, 48, and 72 h, 1 × 10^6^ cells from A549, HepG2, MCF-7, and normal Wi38 lines, either treated or untreated with 100 µg/mL of purified l-glutaminase, was collected. The harvested cells were then resuspended in 10 µM DCFH-DA and incubated at 37 °C for 30 min in the dark. Fluorescence intensity was subsequently measured using a spectrofluorimeter at an excitation wavelength of 485 nm and an emission wavelength of 530 nm. The detected fluorescence intensity is directly proportional to the level of ROS produced.

#### Biochemical parameters

The lipid peroxidation marker malondialdehyde (MDA) and reduced glutathione (GSH) levels were measured using a commercial kit from Cell Signaling, in accordance with the manufacturer’s protocol.

#### Statistical analysis

All the trials were performed in biological triplicate and their means were described with SD (standard deviation). ANOVA (analysis of variance) followed by Tukey´s HSD test was performed in order to detect the significance at *P* < 0.05 between samples. Various letters indicate the significance among samples.

## Results and discussion

### Screening for the potent I-glutaminase producing isolate

Twenty fungal isolates were initially isolated from different soil samples and their capability to grow on selective minimal agar media supplemented with I-glutamine as the sole nitrogen source and pH indicator (phenol red) was examined. Among the developed soil fungal isolates, twelve isolates showed a noticeable intense pink zone with high zone indices in a qualitative examination using GSA medium containing phenol red. Further, the I-glutaminase production was then determined for the selected isolates as mentioned before in Materials and Methods. A harmonic correlation between the zone indices and the enzymatic activity of the I-glutaminase producing fungi was detected.

**Fig. 1:**
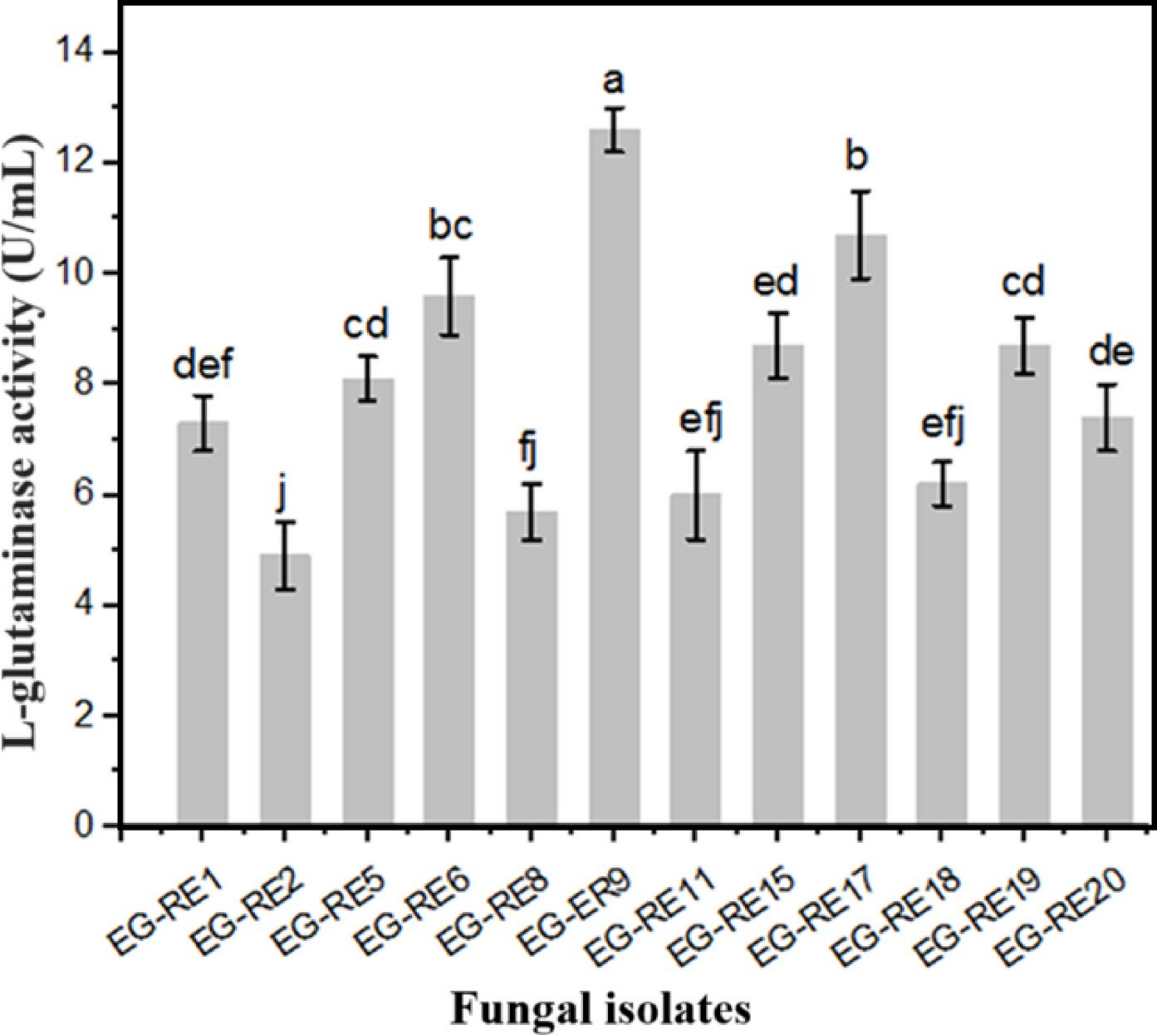
Screening for I-glutaminase production by different fungal isolates. Enzymatic activity was measured at 30 °C using I-glutamine as substrate. Vertical bars presented the mean ± standard deviation (n = 3), various letters represent significantly different at *P* < 0.05.

The results showed that all selected isolates displayed plausible fluctuations in the I-glutaminase production, however, there are a significant difference (*P* < 0.05) in the enzymatic production among the tested isolates which represented by different letter as illustrated in (Fig. 1). The highest I-glutaminase production was determined by the fungal isolate, EG-RE9 (12.6 U/mL). Hence, EG-RE9 was selected in the present work for the further studies.

Similar results showing the potentiality of I-glutaminase production by different fungal isolates and the variation in the enzymatic production among various I-glutaminase producing isolates were observed ^6,48–50^.

### Molecular identification of the potent I-glutaminase producing isolate

**Fig. 2:**
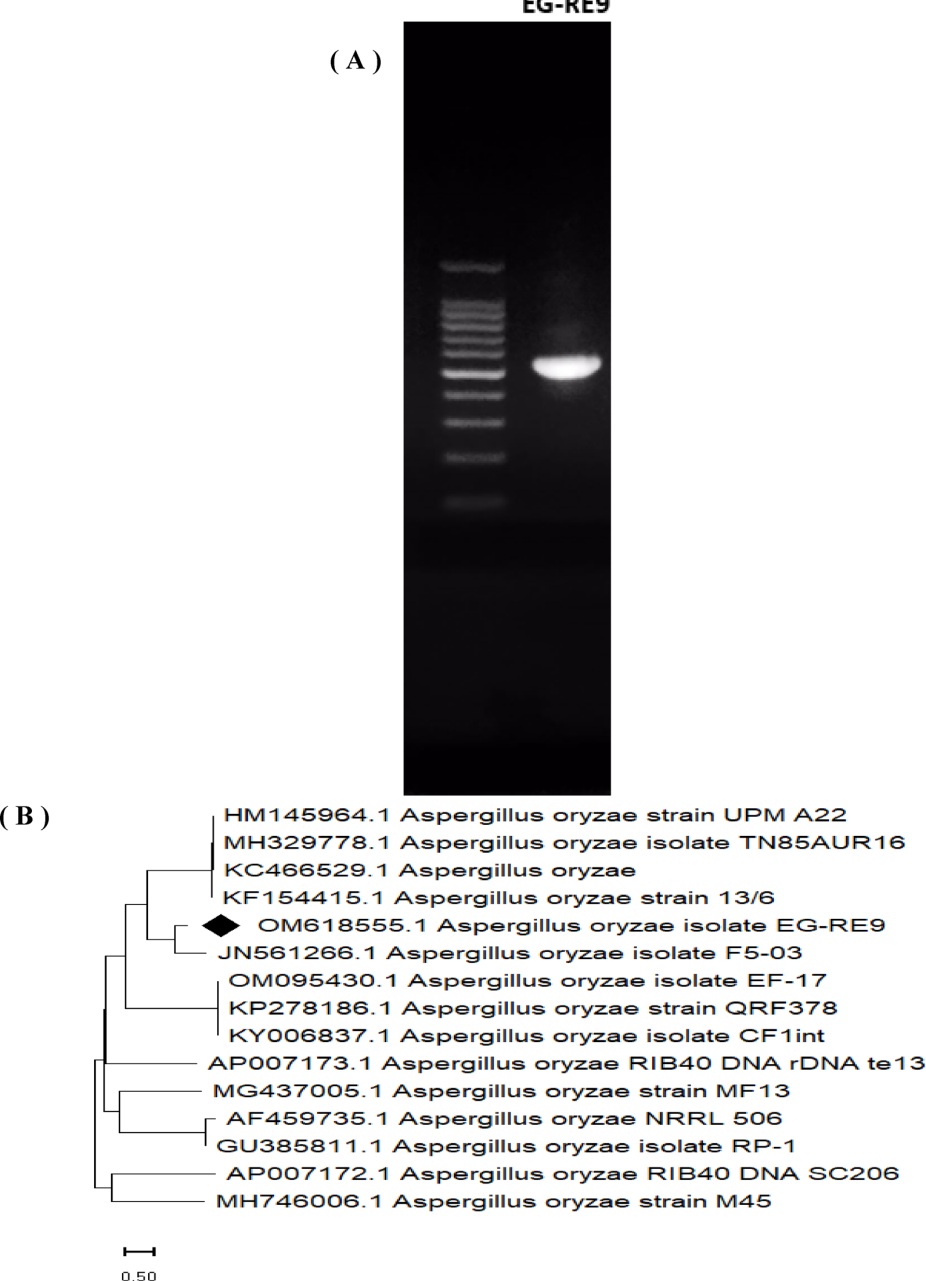
(**A**) Amplicon of ITS-rDNA region from *Aspergillus oryzae* EG-RE9 (**B**) Phylogenetic analysis of *A. oryzae* EG-RE9 sequence and the related GenBank sequences. The tree was performed using the neighbor-joining method with a confidence level of 1000 bootstrap. The ITS-rDNA fragment sequence retrieved from the current study was referred by the symbol ◆.

The promising I-glutaminase producing isolate EG-RE9 was authenticated by the amplification and sequencing of it’s ITS region. The obtained nucleotide sequence was about 597 bp (Fig. 2A) and were harmonized with the reference GenBank nucleotide sequences using BLSAT algorithm tool. The results showed that the fungal isolate EG-RE9 was closely similar to *Aspergillus oryzae* with a 100% similarity percentage*.* The ITS sequence was deposited on the GenBank database with accession number OM618555.1. The phylogenetic tree was concluded using the rDNA sequence and the related sequences through applying the neighbor-joining method with 1000 bootstrap confidence level (Fig. 2B). The genetic identification based on the ITS fragments of the rDNA is a promising tool for the identification of various fungi. In addition, the fluctuations among the ITS sequences were used to construct the phylogenetic analyses ^5^.

### Purification of I-glutaminase

**Fig. 3:**
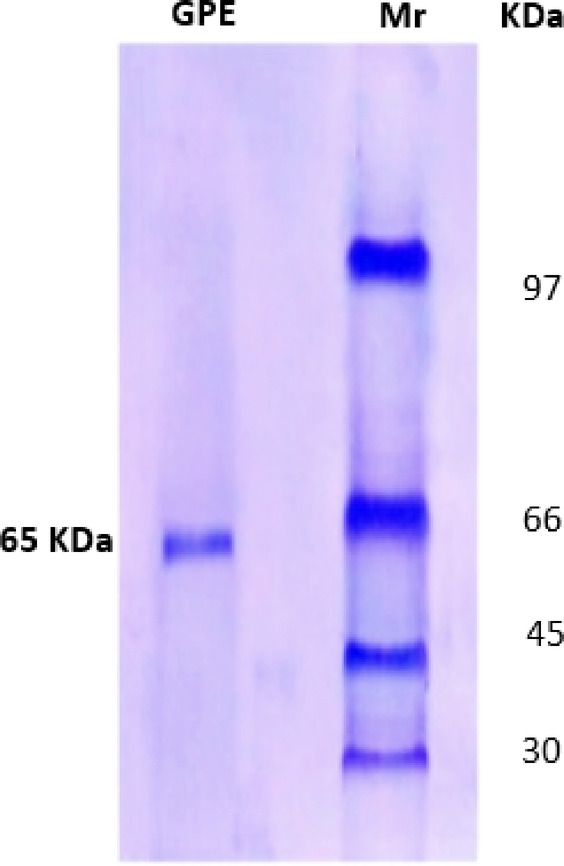
SDS-PAGE electrophoretogram analysis of I-glutaminase from *Aspergillus oryzae* EG-RE9. Lane (Mr) protein ladder (negative control), Lane (GPE) I-glutaminase purified enzyme.

The purification procedure of extracellular I-glutaminase obtained from *A. oryzae* EG-RE9 was performed through salting out, ion exchange and gel filtration chromatography. The overall purification steps of extracellular I-glutaminase are summarized in Table 1. By the last step, the enzyme displayed a specific activity 258.33 (U/mg of protein), with about 215-fold and 36.5% yield. While, the purification fold of the purified I-glutaminase obtained from *Aspergillus* sp. was 36.72 ^51^, *A. oryzae* was 10.2 ^4^, *A. flavus* was 12.47 ^10^ and *A. versicolor* was 2.1 ^6^. The molecular homogeneity of the purified _L_-glutaminase was ascertained by SDS-PAGE analysis with a molecular mass of 65 kDa (Fig. 3). Based on SDS-PAGE analysis, the purified I-glutaminase showed a single band with a molecular mass of 68, 69, 70, and 61.8 kDa for *A. oryzae*, *A. flavus*, and *A. versicolor*, respectively ^4,6,10,50^.

**Table 1:** Sequential steps of the purification profile of *Aspergillus oryzae* EG-RE9 I-glutaminase.

Purification schedule	Total activity (U)	Total protein (mg)	Specific activity (U/mg)	Purification fold	Yield (%)
Crude enzyme	850	710	1.2	1	100
Ammonium sulfate (30–90%)	610	422	1.4	1.17	71.8
DEAE-cellulose	441	30	14.7	12.3	51.9
Sephadex G-200	310	1.2	258	215	36.5

### Biochemical characterization of ***A. oryzae***I-glutaminase

#### Reaction pH, temperature, pH stability, thermostability and substrate affinity

**Fig. 4:**
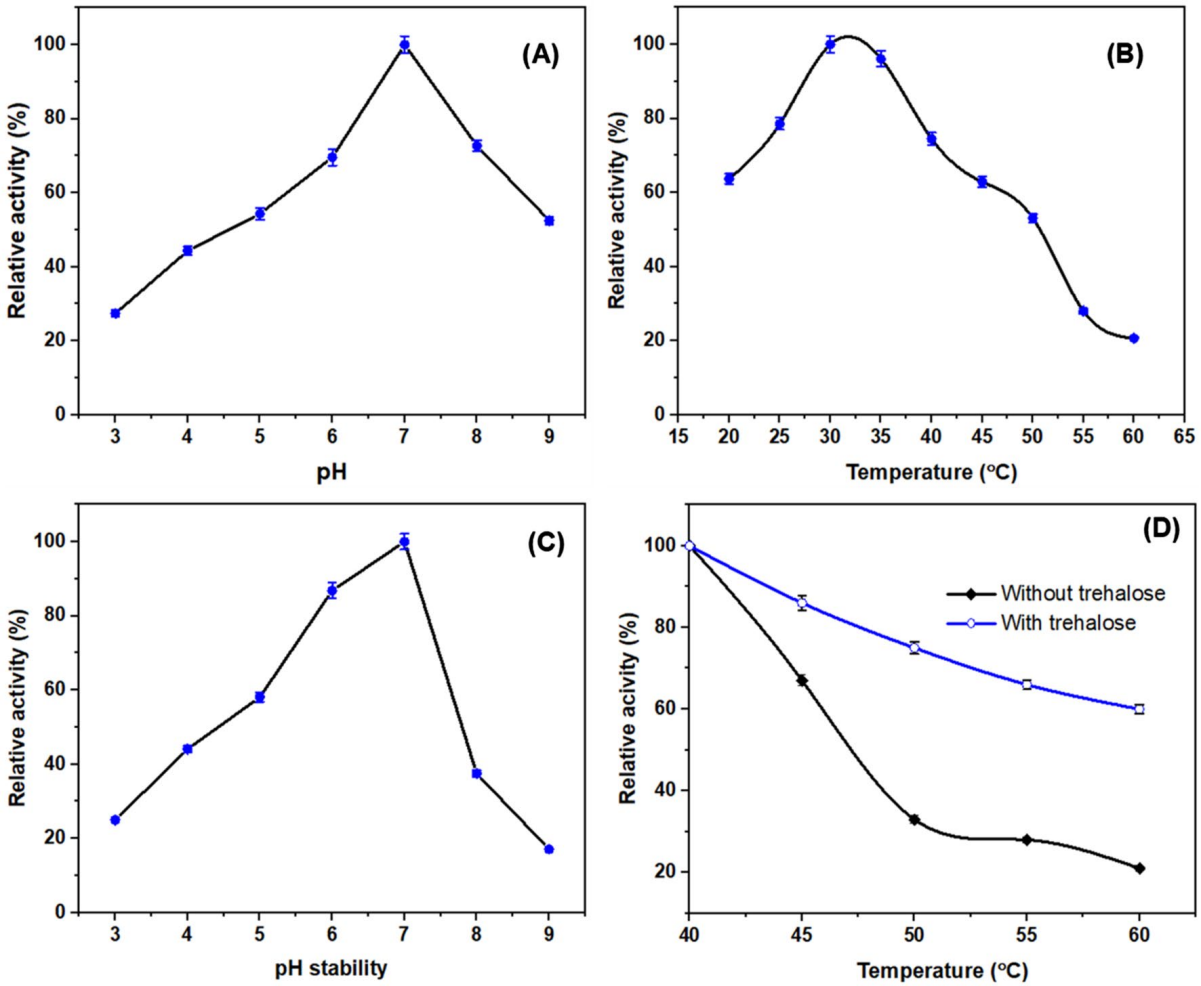
Effect of pH (**A**), and temperature (**B**) on the purified *A. oryzae* I-glutaminase activity. (**C**) pH stability of the purified *A. oryzae* I-glutaminase. (**D**) Effect of 10 mM trehalose on the thermal stability of the purified *A. oryzae* I-glutaminase activity. The enzyme was pre-incubated for 20 min at different temperatures either in the presence or absence of trehalose at 45, 50,55 and 60 °C. Enzyme activity was monitored with the determination assay mentioned above using 1% of I-glutamine. Relative activity of I-glutaminase was determined in relation to the maximum percentage which referenced as 100%, n = 3.

The effect of various pH values was investigated over the range from 3.0 to 9.0 using different buffers (Fig. 4A) in presence of I-glutamine, revealing the pH 7.0 as the choicest pH for maximum I-glutaminase activity. However, the enzyme activity reduced to 27.6% at pH 3.0 and 52.3% at pH 9.0. A strong dramatic reduction in the enzymatic activity was experimentally determined under more acidic and basic pH, however, a mild reduction for alkaline pH was observed. The maximum activity of I-glutaminase from various microbial sources was determined at pH 7.0 ^4,7,50,52^.

At lower and higher pH values, the remarkable reduction could be explained by modifying the surface charge of enzyme, dissociating the subunits or coenzyme, reducing the binding affinity of substrate toward active site, destabilizing the biocatalyst-substrate complex and minimizing the enzyme activity ^5,7^.

The pH stability of I-glutaminase from *A. oryzae* was monitored by pre-incubating at different pH values in the absence of substrate for 1 h, followed by detecting the residual activity based on the standard assay (Fig. 4C). The enzyme lost approximately 73% and 82% of its initial activity at pH 3.0 and pH 9.0, respectively. Similar results have been determined for *Aspergillus versicolor *^6^.

The response of I-glutaminase from *A. oryzae* toward different temperatures (20–60°C) was investigated. The results in Fig. 4B revealed that the highest enzyme activity was observed at 30–35 °C, subsequently a descending order by higher and lower reaction temperature was detected. The obtained temperature/activity profile was in consistence with other investigators ^4,7,10^. Such dramatic reduction in the enzymatic activity could be attributed to the denaturation of enzyme subunits, conformational changes in catalytic active site, rise in the enzyme system inherent energy, unfolding of the tertiary structure, and hence reduce the proper orientation of enzyme for substrate binding ^5,7,33^.

Thermostability of *A. oryzae* I-glutaminase either in presence or absence of 10 mM trehalose was investigated and the results are shown in Fig. 4D. Generally, thermal stability is dramatically decreased with the elevation in the incubation temperature either in the presence or absence of trehalose. However, the presence of an exogenous trehalose in the reaction mixture can improve the resistance of I-glutaminase toward temperature over the biocatalytic activity obtained in the absence of trehalose ^53–57^. Trehalose is a vital osmolytes and effective cytoprotective agent, which enable various fungi to overcome adversity ^57^.

El-Shora and El-Sharkawy ^33^ mentioned the ability to protect the tyrosinase enzyme activity through using of an exogenous substrate in the reaction mixture. Gancedo and Flores ^55,57–59^ suggested that the endogenous and exogenous trehalose is an important way to protect the cell from the outflow of intracellular water and to resist the unfavorable extreme conditions.

**Fig. 5:**
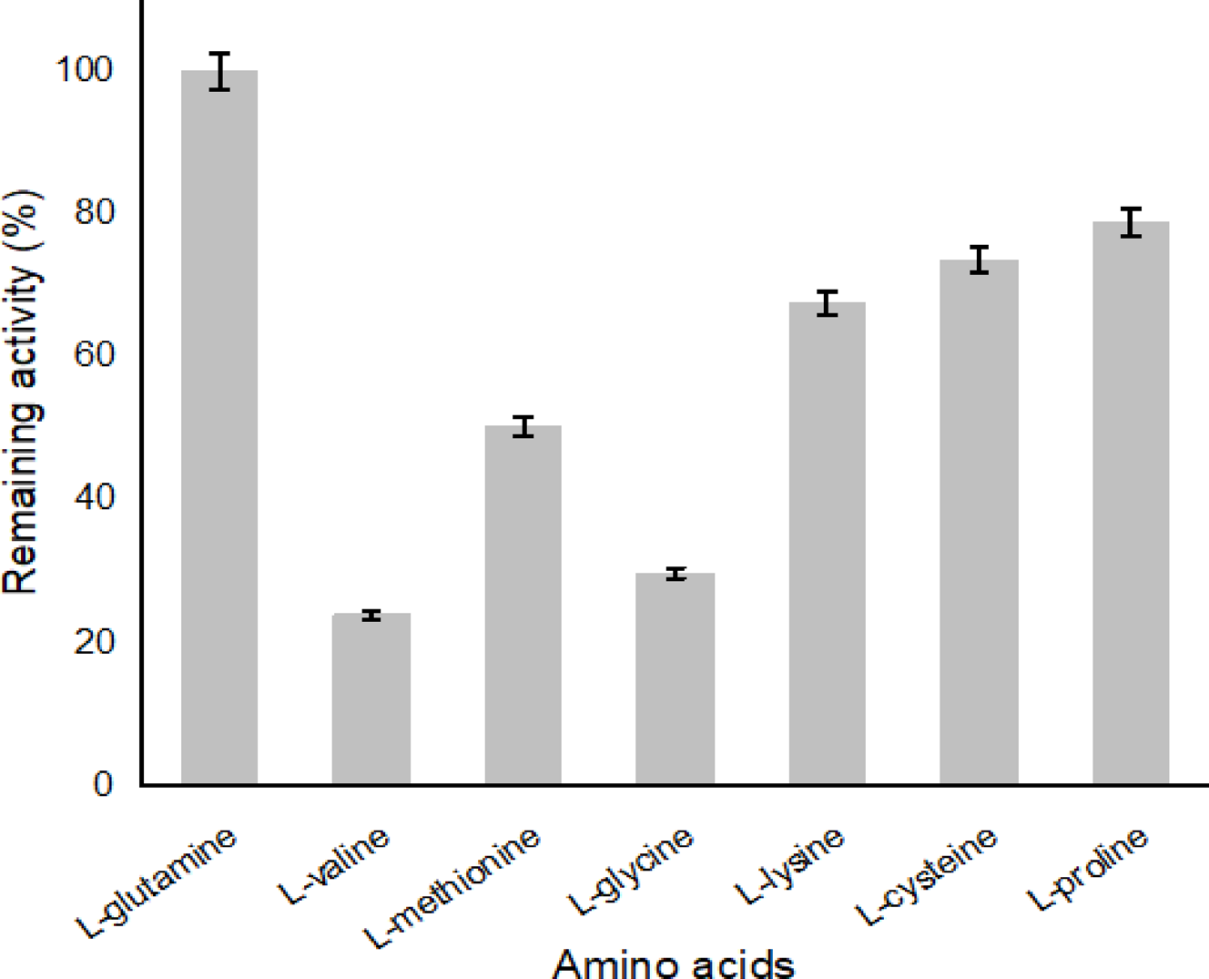
Substrate affinity of the purified *A. oryzae* I-glutaminase activity. Enzyme activity was monitored at standard conditions using 1% of different amino acids substrates. Relative activity of I-glutaminase was determined in relation to the control (I-glutamine, which referenced as 100%), n = 3.

*A. oryzae* I-glutaminase was evaluated using different amino acids in the standard conditions relative to the control (_L_-glutamine, which referenced as 100%). The enzyme exhibited a plausible activity towards _L_-glutamine, _L_-cysteine, _L_-proline and _L_-lysine (Fig. 5). The enzyme lost approximately 70% and 75% of its initial activity when _L_-glycine and L-valine were used as substrates, respectively. The maximum activity of I-glutaminase was authenticated by using _L_-glutamine as a standard substrate ^7,10,50^.

#### Susceptibility towards different metals ions

**Fig. 6:**
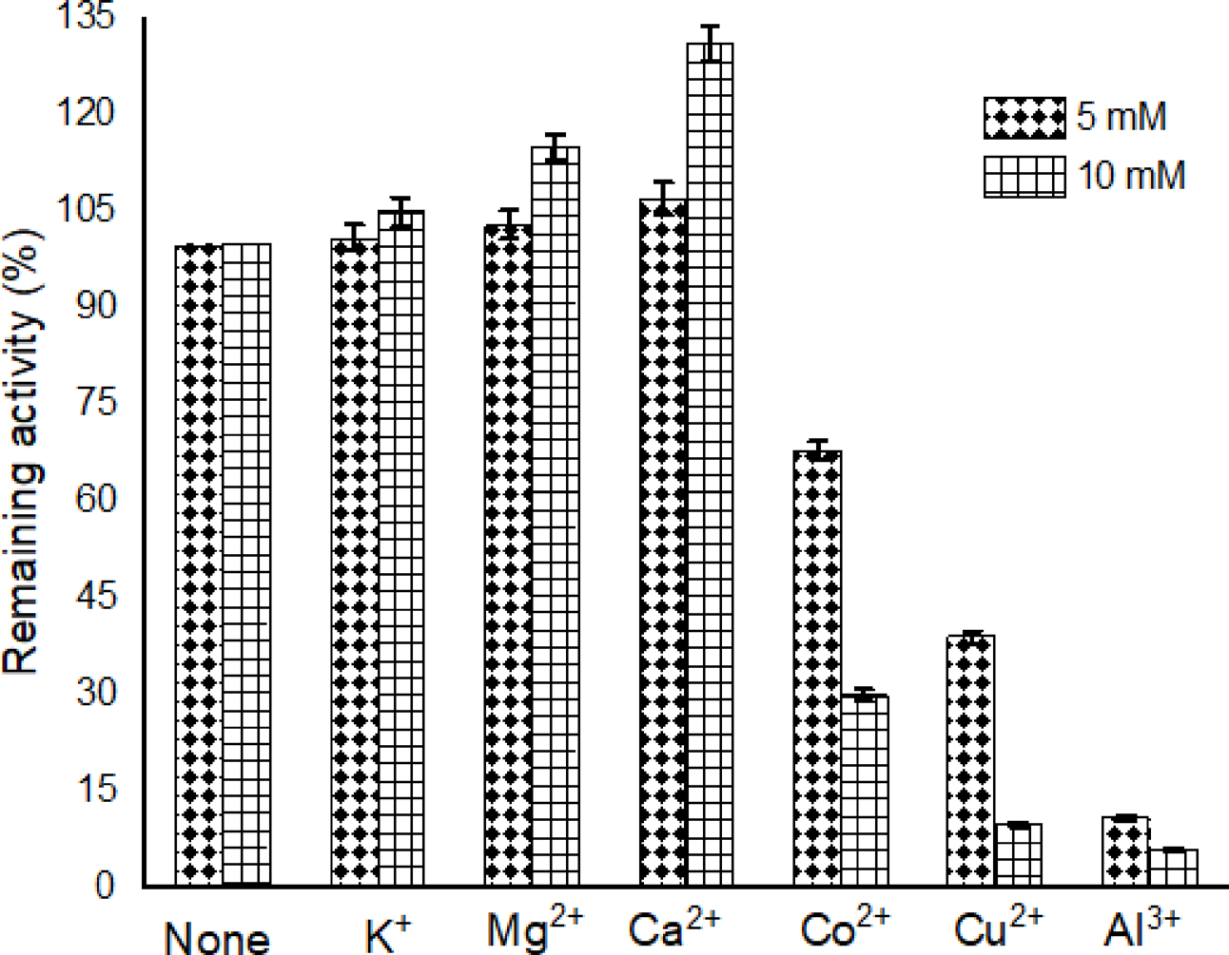
Effect of different metals ions on the purified *A. oryzae* I-glutaminase activity. Enzyme activity was monitored at standard conditions using 1% I-glutamine. Relative activity of I-glutaminase was determined in relation to the control (without any additives), n = 3.

The influence of different metals ions on the purified *A. oryzae* I-glutaminase has been investigated by pre-incubating the biocatalyst for 1 h at 35 °C in a final concentration of 5 mM and 10 mM. The enzymatic activity was assayed in standard conditions. The results depicted in Fig. 6 showed that the relative activity of the purified I-glutaminase was increased to the extent of 100–120% with the incorporation of K^+^, Mg^2+^, and Ca^2+^ at 5 mM and 10 mM. However, the incorporation of Co^2+^, Cu^2+^, and Al^3+^ cations displayed a remarkable reduction by 68%, 39%, and 11% at 5 mM and 30%, 10%, and 6% at 10 mM of the enzymatic residual activities, respectively. Previous studies described that the metals ions displayed a negative or positive effect on the bio-catalytically catalyzed reactions. The enzymatic activity of I-glutaminase from the recombinant AYA 20–1 was stimulated in the presence of Co^2+^, K^+^, Na^+^ and Ca^2+^ ^49^. Conversely, Ca^2+^, Mg^2+^, Hg^2+^, Zn^2+^, Cu^2+^ and Ba^2+^ did not display any positive effect on the residual activities of the biocatalyst ^10,60^.

The stimulatory effect on the I-glutaminase activity by the addition of metal cations may be attributed to their protection of the biocatalyst against thermal denaturation and proteolysis as well as the stabilization of the stero-structure of the enzyme active site by maintaining the tertiary and quaternary structures of the enzyme molecule. The stimulatory effect of Ca^2+^ may have been as a result of the fact that some microbial extracellular enzymes require Ca^2+^ for their activity and stabilization ^6,10^.

#### Susceptibility towards active group reagents

**Fig. 7:**
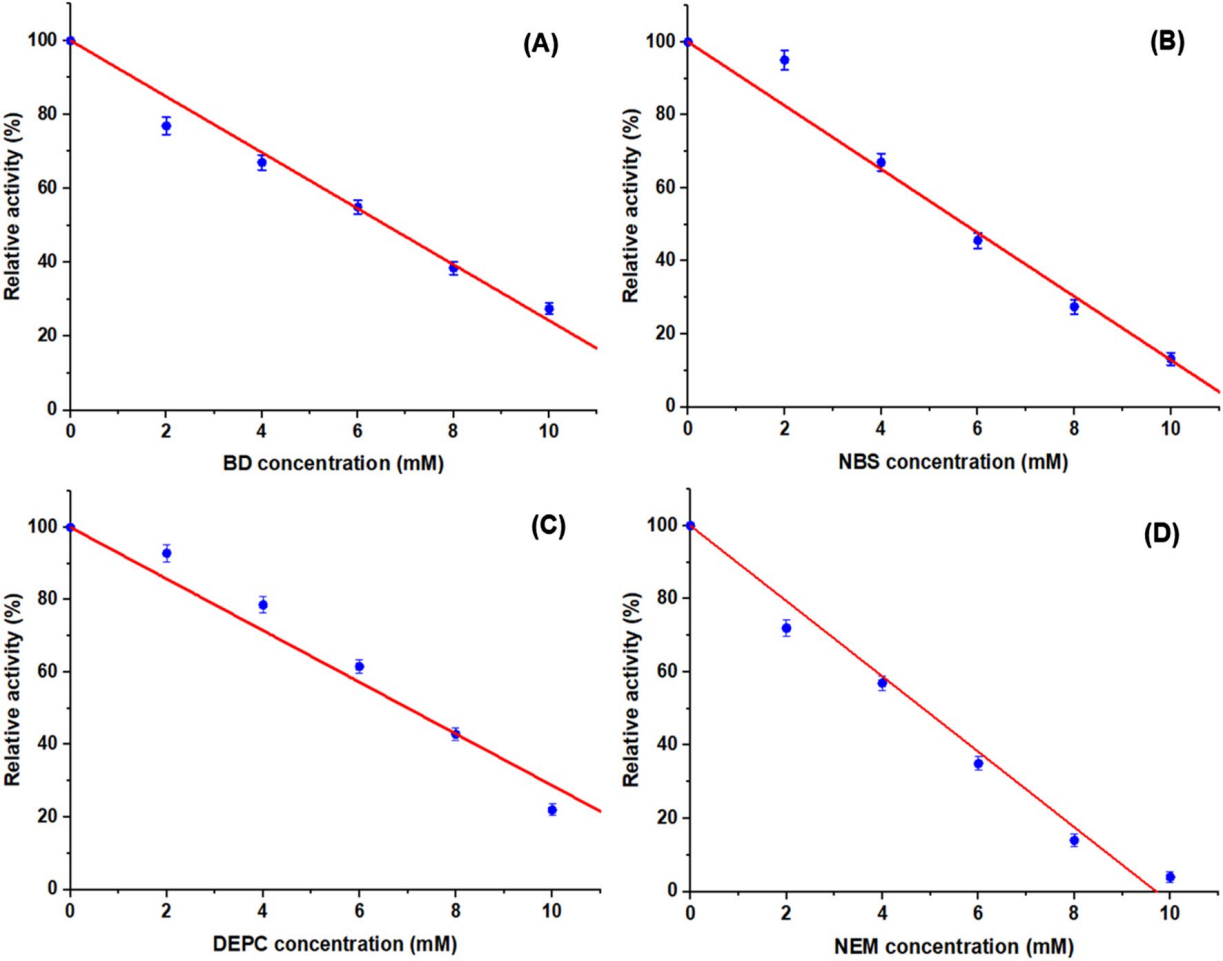
Effect of some group specific modifiers on the activity of I-glutaminase from *A. oryzae* EG-RE9. The activity in the absence of agents was considered as 100% and the relative activity was represented as % of the control.

Butanedione (BD), N-bromosuccinimide (NBS), diethyl pyrocarbonate (DEPC), and N-ethylmaleimide (NEM) are reagents used to investigate the involvement of arginyl, tryptophanyl, histidyl, and cysteinyl residues, respectively, in enzyme catalysis ^61,62^. In order to detect the unique amino acids in the *A. oryzae* I-glutaminase-active sites with their essentiality in the enzymatic catalysis, the enzyme was incubated with the above suicide inhibitors (Fig. 7).

The biocatalyst was pre-incubated with such group specific modifiers at concentrations between 2 and 10 mM for 20 min, and then supplemented with I-glutamine. The relative enzyme activity was estimated, related to the controls without any agent. The purified I-glutaminase activity was dramatically inhibited in the presence of BD, NBS, DEPC, and NEM, suggesting the essentiality of arginine, tryptophan, histidine, and cysteine residues in the catalysis process of I-glutaminase, respectively. The IC_50_ of BD, NBS, DEPC, and NEM for *A. oryzae*_L_-glutaminase were 6.6, 5.7, 7.0, and 4.8 mM, respectively. Such findings are in partially consistent with those recorded by the recent works ^5,6,10,33^.

#### Kinetic parameters

**Fig. 8:**
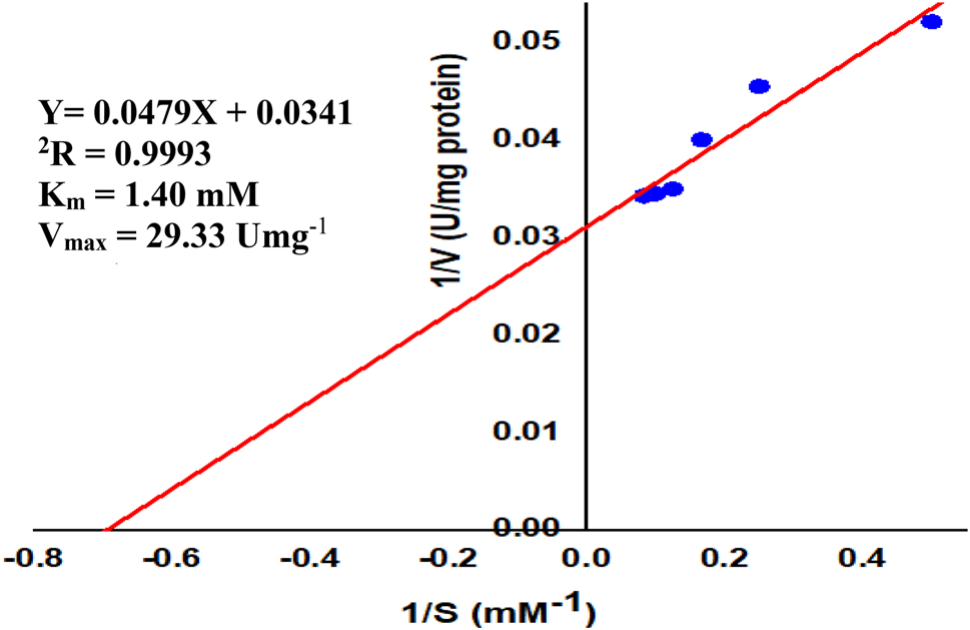
Kinetic parameters of the purified I-glutaminase from *A. oryzae* EG-RE9. The enzymatic activity was determined at I-glutamine concentrations between 0.25 mM and 12 mM under optimal assay conditions.

The kinetic properties of the purified *A. oryzae* I-glutaminase were assayed in optimal conditions and are illustrated in Fig. 8. The V_max_ and K_m_ values were calculated and they were 29.33 U/mg protein and 1.40 mM, respectively. The lower K_m_ value of I-glutaminase indicates higher affinity of the enzyme for _L_-glutamine. The maximum I-glutaminase activity from *Penicillium brevicompactum* NRC 829 was determined at K_m_ of 1.66 mM as reported by Elshafei A. M. et al., ^63^.

From the Lineweaver-Bürk plot using different concentrations of I-glutamine, V_max_ of 95 U/mg protein and K_m_ of 50 mM were reported by^4^. Abu-Tahon and Isaac ^10^ reported that the *A. flavus* I-glutaminase displayed a typical Michaelis–Menten behavior with K_m_ of 4.5 mM and V_max_ of 20 U/mg protein. Thus, I-glutaminase exhibited the advantages of lower K_m_ and higher V_max_, compared to the values of these two parameters from other sources.

### Biotechnological applications of I-glutaminase

#### Production of I-theanine

**Fig. 9:**
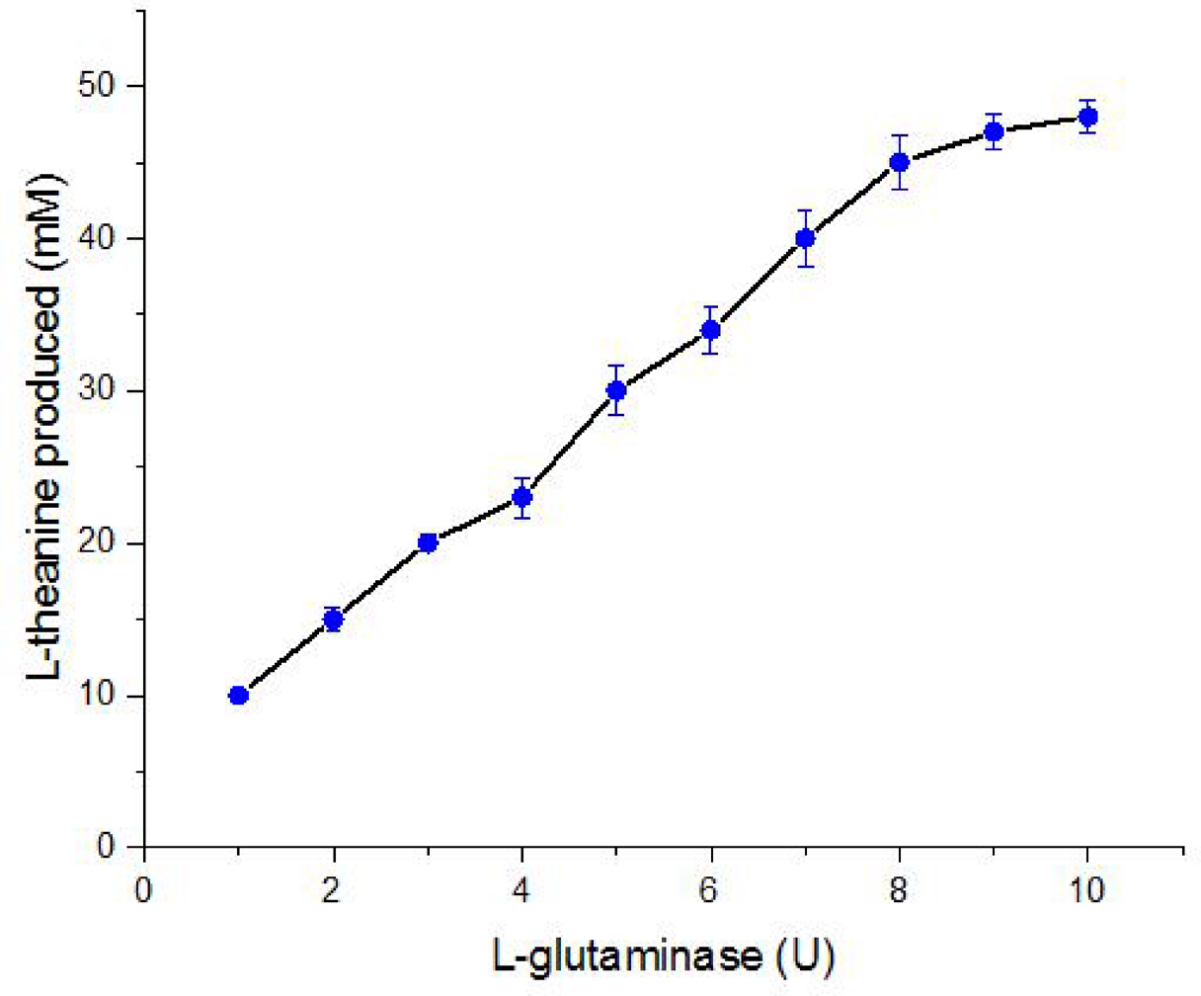
Production of I-theanine using various concentrations of I-glutaminase obtained from *A. oryzae* EG-RE9. The reaction mixture contains 1.3 M ethylamine, 0.01 M ATP, 0.05 M MnCl_2_, 0.2 M I-glutamate and I-glutaminase (1–10 units) in phosphate buffer (pH 7.0). The reaction was incubated at ambient temperature for 48 h and 150 rpm.

The I-theanine produced using the purified *A. oryzae* I-glutaminase was monitored with various amounts of I-glutaminase (1–10 units). The results depicted in Fig. 9 clearly illustrates that the concentration of l-theanine was increased by increasing the units of I-glutaminase used in the reaction mixture, reaching the maximum level at 10 units of the purified enzyme. These results are in harmony with those of recent paper ^12^ who reported that presence ethylamine and l-glutamine enable the synthesis of l-theanine and l-glutamic acid by *Trichoderma koningii* I-glutaminase.

#### Deamidation degree of glutelin from ***Zea*** and Rice using I-glutaminase

**Fig. 10:**
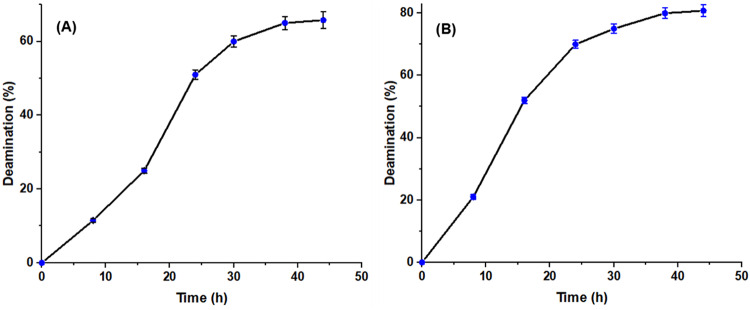
Deamidation degree of *Zea* (**A**) and rice (**B**) glutelin using *A. oryzae* I-glutaminase at different time intervals. The glutelin deamidation reaction was performed in phosphate buffer including 10 mg/ml glutelin and I-glutaminase (0.5 unit) and was incubated at 30 °C for 0–60 h.

The deamidation of *Zea* and rice glutelin using I-glutaminase was performed through sequential steps namely: defatting of flour, preparation of flour glutelin and the deamidation process. Deamidation degree of *Zea* and rice glutelin (Fig. 10) was increased with increasing the time of reaction, recording approximately 65% and 83% after 44 h of enzymatic treatment, respectively. This possibly because the peptide bonds are amide bonds, they can also be cleaved during the reaction, producing deamidated peptides with a lower Mw. Moreover, the rise in negative charges may enhance electrostatic repulsion between molecules, thereby reducing both intra- and intermolecular hydrogen bonding. This, in turn, could weaken molecular aggregation and lead to an increase in protein solubility in water. The rice and wheat glutelin deamidation have been recorded by purified glutaminase from *Chryseobacterium proteolyticum *^23,64^.

Wong et al.^45^ reported that the level of deamidation degree is an important indicator for measuring the needed functional and conformational characteristics in the deamidated food proteins. Generally, the glutamine residues may be surrounded by the glutelin molecules aggregates as a result of their powerful hydrophobic interaction, resulting in a reduction of the deamidation degree in the initial stage of enzymatic reaction. However, the further progress of I-glutaminase reaction is possibly results in an increase in the glutamine modifying action through reaching the buried sites^24,58,65^.

#### Production of l-glutamic acid

**Fig. 11:**
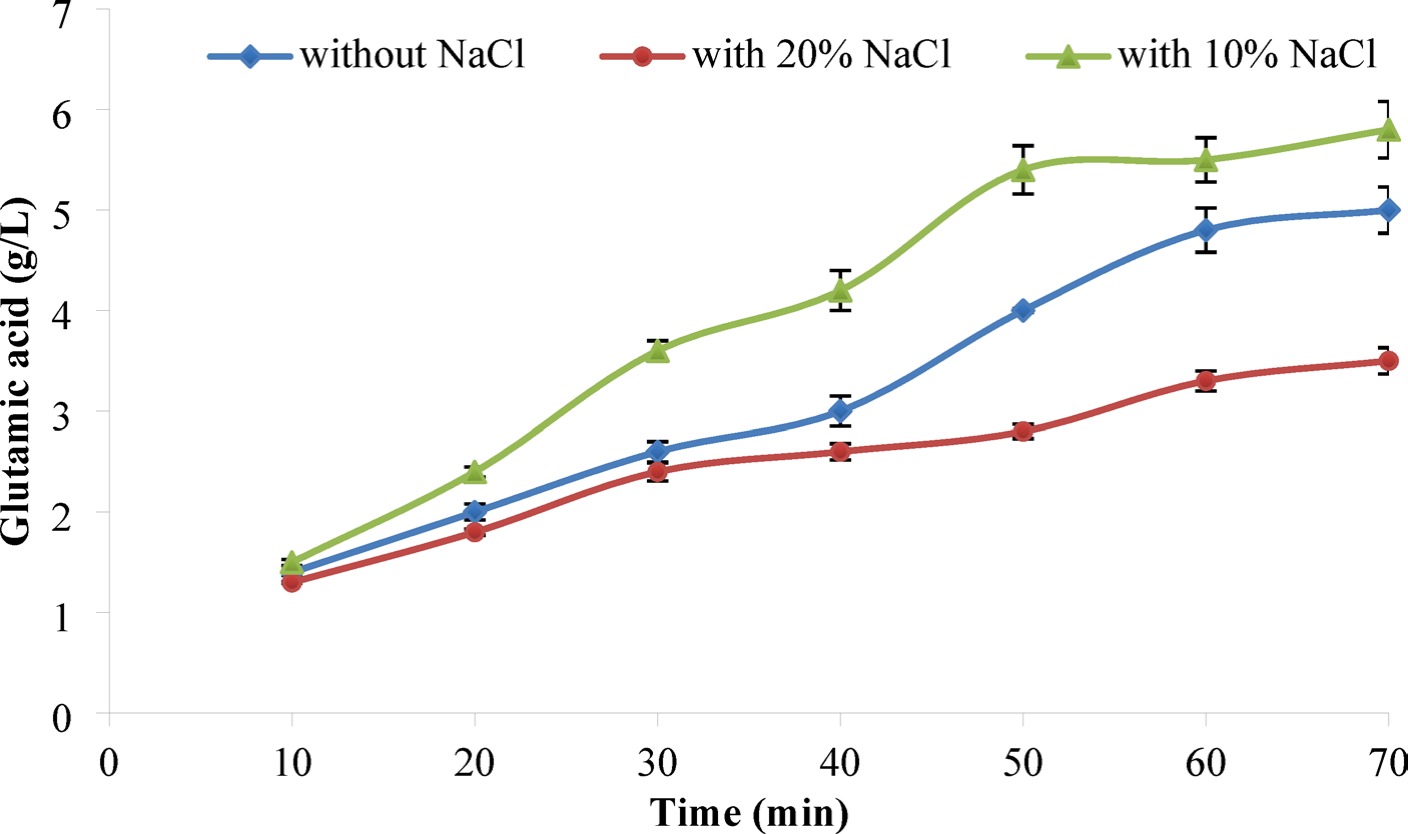
l-glutamic acid production using *A. oryzae* l-glutaminase at different time intervals in the presence or absence of 10% and 15% NaCl. The reaction was performed at 30 °C for 70 h.

The concentration of l-glutamic acid resulted from soybean by using l-glutaminase is illustrated in Fig. 11. _L_-glutamic acid concentration increased gradually with the increase in the reaction time. The resulted l-glutamic acid in the presence of NaCl with a final concentration 10% is preferred over the 15% concentration for the hydrolysis process. Bülbül and Karakuş ^66^ observed that l-glutaminase activity from *Hypocrea jecorina* increased with the addition of 5% salt to the culture medium, whereas higher salt concentrations resulted in reduced enzyme activity. Similarly, Weingand-Ziad et al. ^67^ investigated the impact of salt concentrations ranging from 0 to 20% on l-glutamic acid production by *Lactobacillus rhamnosus*, identifying 2.5% as the optimal concentration. In addition, studies by Parasanth-Kumar et al. ^68^ and Mostafa et al. ^69^ indicated that l-glutaminase activity generally increases in presence of NaCl at concentration not more than 10%.

#### The antitumor activity

**Fig. 12:**
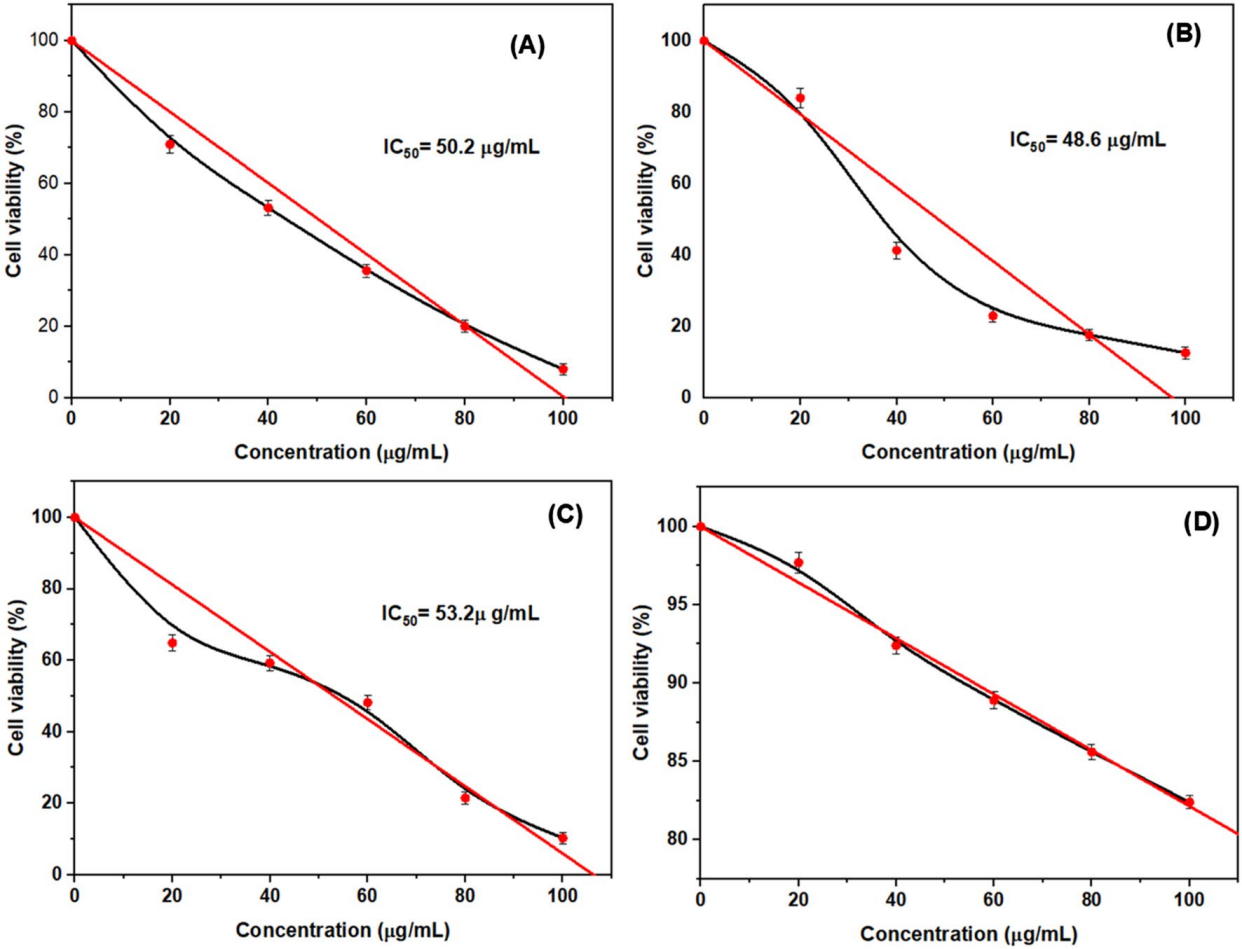
Cytotoxic effect of I-glutaminase toward (**A**) lung cancer (A549), (**B**) liver cancer (HepG2), (**C**) breast cancer (MCF-7) cell lines and (**D**) human normal cell line (Wi 38) as control. The cells were subject to various concentrations of the enzyme for 24 h at 37 °C. Cell viability was measured using MTT assay.

MTT assay was used to evaluate the antitumor activities of the purified I-glutaminase over a concentration range 20–100 µg/mL and the results were illustrated in Fig. 12 A–D. The results showed a continuous reduction in cell viability of lung (A549), Liver (HepG2), breast (MCF-7) tumor cell lines with increasing the concentration of I-glutaminase. The results also revealed that the enzyme exhibited antitumor activity against the tested A549, HepG2, MCF-7 cancer cell lines with IC_50_ values of 50.2, 48.6 and 53.2 µg/mL, respectively; comparing to the standard drug doxorubicin which has IC_50_ values of 2.9, 13.0, and 2.96 µg/mL for A549, HepG2 and MCF-7 cell lines, respectively. I-glutaminase did not display remarkable toxicity against normal cell lines (Wi 38) comparing to doxorubicin and IC_50_ for normal cell lines was > 100 µg/mL of I-glutaminase. Similar findings has been reported by recent articles ^10,66^ who found the antitumor activity of the purified I-glutaminase against different tested cell lines.

#### The intracellular ROS generation and biochemical parameters

Dichlorodihydrofluorescein diacetate (DCFH-DA) is widely utilized for detecting reactive oxygen species (ROS) and evaluating oxidative stress. After entering the cell, intracellular esterases cleave the acetyl groups from DCFH-DA, producing the non-fluorescent compound DCFH. This molecule can then be oxidized by various ROS, including hydrogen peroxide, hydroxyl radicals, and peroxynitrite, as noted by Liu J. et al., ^70^. The oxidation of DCFH generates dichlorofluorescein (DCF), which exhibits green fluorescence with an excitation wavelength of 485 nm and an emission wavelength of 530 nm.

**Fig. 13:**
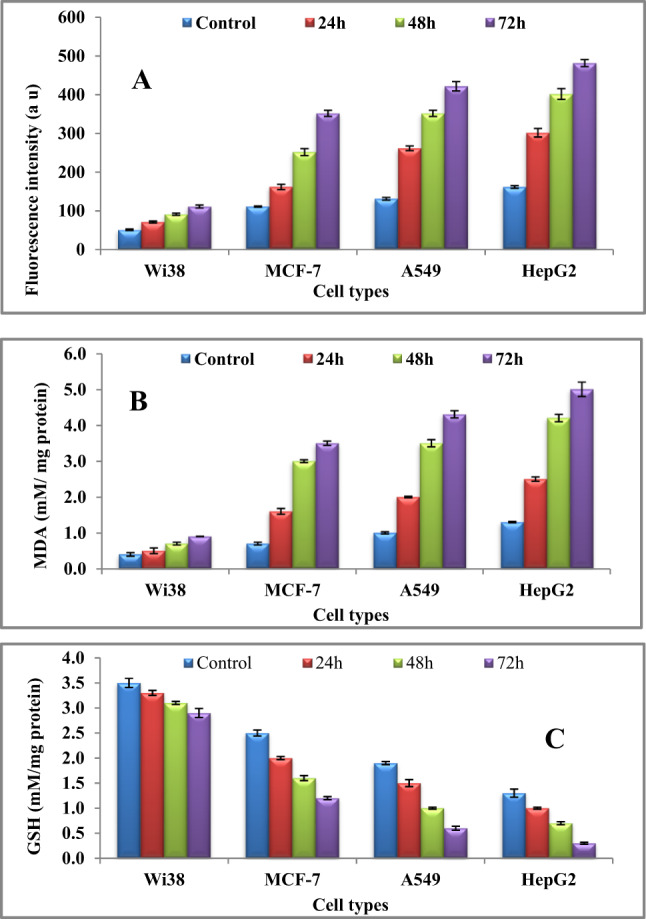
(**A**) Fluorescence intensity of ROS, (**B**) induction of MDA level and **(C)** reduction of GSH content in Wi38 (normal), MCF-7, A549, and HepG2 cells after treatment with l-glutaminase.

In this study, treatment of various cancer cell lines with purified l-glutaminase led to a marked accumulation of ROS (Fig. 13A) compared to the normal Wi-38 cells. Moreover, l-glutaminase exposure resulted in elevated MDA levels (Fig. 13B) and a time-dependent decrease in GSH content (Fig. 13C). The increase in ROS levels as indicated by the detected fluorescence intensity along with lipid peroxidation and diminished antioxidant defenses, contributes to cell death through oxidative stress ^71^. The rise in ROS levels leads to the oxidation of lipids, a process known as lipid peroxidation (LPO), which results in the formation of MDA. During LPO of membrane lipids, membrane depolarization and the inactivation of membrane-bound enzymes take place, significantly impacting membrane transport and altering cell shape ^72^.

The redox imbalance signifies a disruption in the balance between ROS generation and elimination, ultimately leading to macromolecular damage ^73^. ROS production has been strongly linked to the induction of apoptosis and reduced survival of cancer cells ^74^. By interacting with cellular proteins, lipids, and DNA, ROS induce oxidative stress, which in turn activates both extrinsic and/or intrinsic apoptotic pathways ^75,76^.

Consequently, ROS are regarded as promising and effective targets for cancer therapy ^77^. Glutamine-deprivation therapy using l-glutaminase, which hydrolyzes l-glutamine into l-glutamic acid and ammonia, selectively inhibits tumor growth by blocking de novo protein synthesis and elevating ROS levels such as superoxide through oxidative stress, thereby promoting cancer cell death ^78–80^. Complete tests should be conducted in future work, including DAPI staining, Real-time PCR (qRT-PCR), Annexin V/PI assay, intracellular reactive oxygen species (ROS) assay, autophagy staining, and wound healing assay (scratch assay).

## Conclusion and future perspectives

In the present work, the production of I-glutaminase by *Aspergillus oryzae* EG-RE9 was investigated. The enzyme was purified and its biochemical properties were evaluated. The purified *A. oryzae* l-glutaminase displayed significant practical applications in l-theanine and l-glutamic production, deamidation of water insoluble glutelin as well as antitumor activity against different tested tumor cells. Therefore, l-glutaminase from *A. oryzae* can perform a potential role in different food products industries and in targeting various cancer cells. Fungal l-glutaminases have engrossed great importance because of their wide and notable industrial applications. Hence, the production of unique catalytically-effective fungal l-glutaminase is significantly desired. Further research is vital for optimizing the production bioprocess of fungal l-glutaminases, and understanding their structural properties, biological roles and biotechnological applicability.

## Supplementary Information

Below is the link to the electronic supplementary material.

Supplementary Material 1

## Data Availability

The data sets used and analyzed during the current study are available from the Gharieb S. El-Sayyad (corresponding author) upon reasonable request.
