# Supplementary figures and images for: Purification and biochemical characterization of l-glutaminase from Aspergillus oryzae with potential biotechnological applications in synthesis of l-theanine and as antitumor agent
Source: Sci Rep. 2025 Oct 21;15:36511. doi: 10.1038/s41598-025-21904-8 (PMC12540682; doi:10.1038/s41598-025-21904-8)

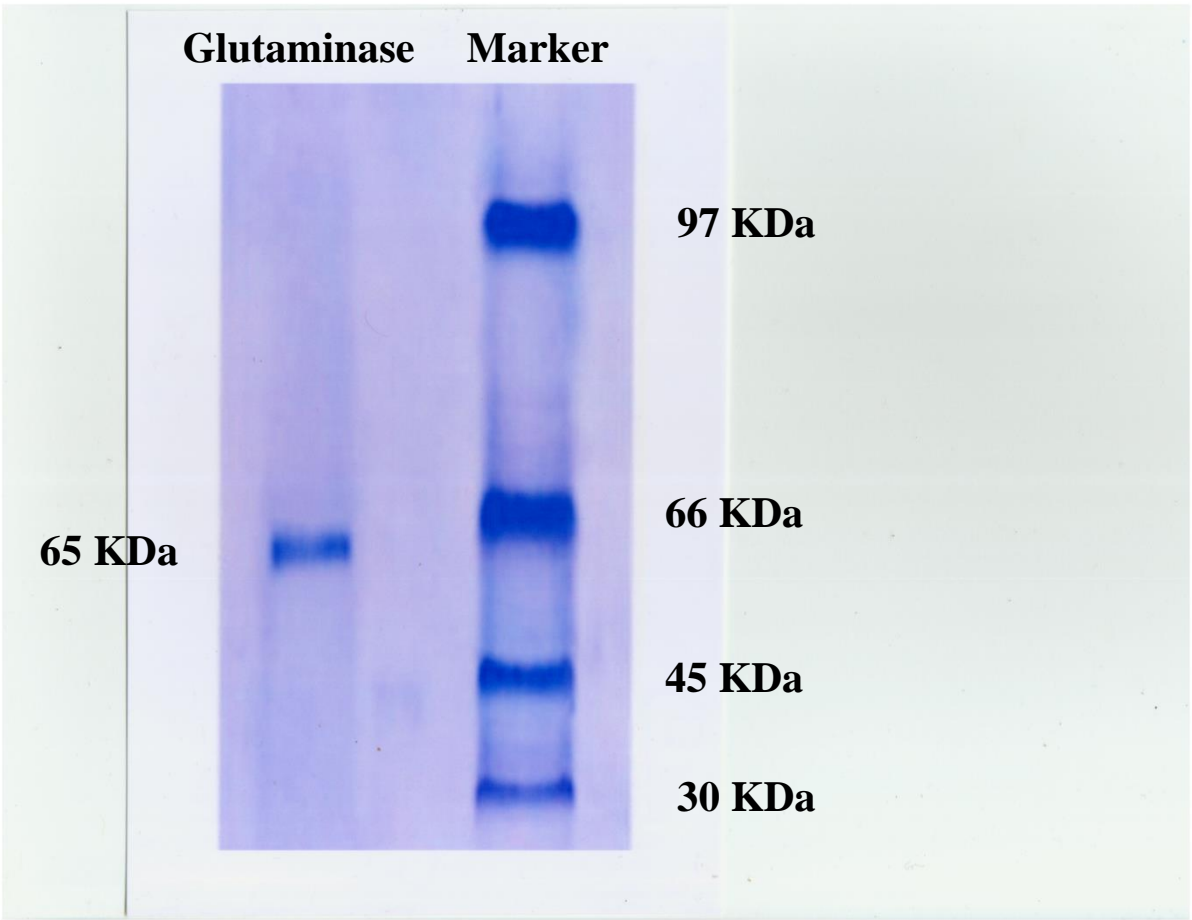

Supplement: Supplementary file 1 — Supplementary Material 1 [file 41598_2025_21904_MOESM1_ESM.pdf]
